# Purification of viable peripheral blood mononuclear cells for biobanking using a robotized liquid handling workstation

**DOI:** 10.1186/s12967-019-2125-7

**Published:** 2019-11-12

**Authors:** Luigi Coppola, Giovanni Smaldone, Alessandra Cianflone, Simona Baselice, Peppino Mirabelli, Marco Salvatore

**Affiliations:** IRCCS SDN, Naples Via Emanuele Gianturco, 113, 80143 Naples, Italy

**Keywords:** Laboratory automation, Biobanks, Samples processing, White blood cells, Diagnostics

## Abstract

**Background:**

The purification of peripheral blood mononuclear cells (PBMCs) by means of density gradient (1.07 g/mL) centrifugation is one of the most commonly used methods in diagnostics and research laboratories as well as in biobanks. Here, we evaluated whether it was possible to set up an automated protocol for PBMC purification using a programmable liquid handling robotized workstation (Tecan, Freedom EVO 150). We selected a population composed of 30 subjects for whom it was possible to dispose of two ethylenediaminetetraacetic acid (EDTA) vacutainer tubes containing unfractionated peripheral blood. The purification of PBMCs was performed in parallel using automated and manual workflows.

**Results:**

An automated workflow using the Freedom EVO 150 liquid handling workstation was generated for the isolation of PBMCs. This protocol allowed blood dilution in Dulbecco’s phosphate-buffered saline (DPBS), stratification onto the density gradient, and the collection of PBMC rings after centrifugation. The comparison between the automated and manual methods revealed no significant differences after separation in terms of total mononuclear cell enrichment, red blood cell contamination, or leucocyte formula, including the percentage of lymphoid subpopulations as B, T and natural killer (NK) lymphocytes.

**Conclusions:**

Our results show that it is possible to set up an automated protocol for the isolation of PBMCs using a robotized liquid handling workstation. This automated protocol provided comparable results to the routinely used manual method. This automatic method could be of interest for those working in biobanking or industries involved in diagnostics and therapeutics field, to avoid operator-dependent errors as well as procedures standardization.

## Background

The foundation of research biobanks after completion of the human genome project has been a critical step for entry in the postgenomic era. Indeed, biobanks provide high-quality samples for the setting up of research protocols dedicated to the translation of important discoveries coming from basic research laboratories to clinical practice [1]. One example of their usefulness comes from the generation of The Cancer Genome Atlas (TCGA) [2]. Because of human specimens with associated clinical data provided by biobanks, it is possible to analyze large cohorts of over 30 tumors with high-throughput genome sequencing. The TCGA led to the identification of several novel molecular alterations in cancer, allowing the classification of tumor subtypes according to distinct genomic alterations and, consequently, a precision medicine approach for patient care [3]. Despite the general consensus regarding the critical role of biobanks in modern research and the growing number of newly founded institutional biobanks, there is a high level of heterogeneity between biobanks, with particular reference to the procedures used for sample processing [4]. In 2018, the International Standardization Organization (ISO), in association with international biobank scientific organizations, such as the International Society for Biological and Environmental Repositories (ISBER) [5] and the European Biobanking and BioMolecular Resources Research Infrastructure (BBMRI) [6], provided the ISO/DIS 20387 standard [7], which is specifically dedicated to the biobanking process. The aim of the ISO20387 is to standardize the processing of biosamples across countries by defining important requirements for correct sample processing. In this context, our work aimed to contribute to the standardization of sample processing by presenting an innovative automatic workflow setup on the Freedom EVO 150 automatic liquid handling workstation. To date, different companies, such as Hamilton (https://www.hamiltoncompany.com), Qiagen (https://www.qiagen.com/us/products/instruments-and-automation/?akamai-feo=off), Brooks Life Sciences (https://www.brookslifesciences.com/about-brooks-life-sciences) and Tecan (https://www.tecan.com/), provide solutions for the customization of automatic procedures for biological sample processing. Due to their versatility, automated liquid handling systems are commonly used for different laboratory applications [8–10], particularly when the pipetting steps must be as accurate and precise as possible, as in the cases of biopharmaceutical products preparation [11, 12], serial dilutions, and reagent transfers [13, 14]. In this paper, we propose these complex technological systems for the automatic purification of peripheral blood mononuclear cells (PBMCs) from whole blood collected in ethylenediaminetetraacetic acid (EDTA) tubes. Because the density gradient purification of PBMCs is a procedure with high operator-dependent variability, we believe that the methodology presented in this paper could be useful for the standardization of PBMC isolation in biobanking.

## Results

### Set-up of automatic isolation of PBMCs

**Fig. 1:**
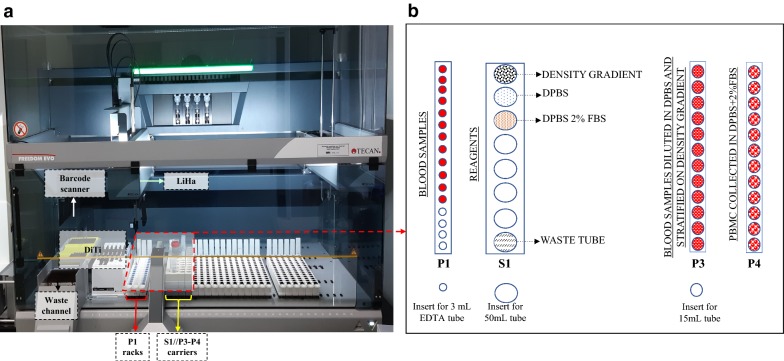
Workstation setup. **a** Liquid handling workstation. **b** Tube rack workstation setup. The P1 rack contains centrifuged PB (Peripheral Blood) in BD vacutainer EDTA tubes. The S1 rack holds 50 mL conical tubes containing density gradient media, DPBS, DPBS + 2% FBS and an empty tube for waste. P3 contains the 15 mL conical tubes where the DPBS-diluted blood will be stratified onto 3 mL of the previously dispensed density gradient media. After centrifugation, the 15 mL conical tubes containing the stratified PB will be relocated to rack P3, and PBMCs will be transferred and diluted in the 15 mL conical tubes located in rack P4 containing 5 mL of DPBS + 2% FBS solution. *DiTi* disposable tips; *LiHa* liquid handling arms

To improve our biobank workflow in isolating PBMCs, we developed an automated method for avoiding operator-dependent variability. Therefore, we established an automatic script to be applied on the Tecan Freedom EVO 150 liquid handling workstation (Fig. 1). The aim was to reproduce all steps for PBMC isolation from peripheral blood in a similar fashion to that of the manual protocol. Figure 1 shows the setup of the workstation. Mononuclear cells were purified from the cellular pellet contained in 3 mL EDTA blood vacutainer tubes (Becton–Dickinson, Ref. 364664) that were previously processed for plasma separation. The pipetting robot Freedom EVO 150 uses a customized human PBMC isolation script where the blood tubes are the first barcode identified. Then, the software converts the location of the blood tubes into X, Y, and Z vectors, which guide the tips of the pipetting arm to dip precisely into the positions set. The positioning of vacutainer tubes is performed in strip rack P1, as depicted in Fig. 1. The strip rack S1 is used for allocating into 50 mL conical tubes the reagents necessary for the entire procedure according to the following scheme and as represented in Fig. 1, rack S1 from the top: (1) density gradient (HiSep™ LSM1077) solution; (2) DPBS; (3) DPBS supplemented with 2% FBS (FBS); and (4) an empty tube for discarding liquid waste.

**Fig. 2:**
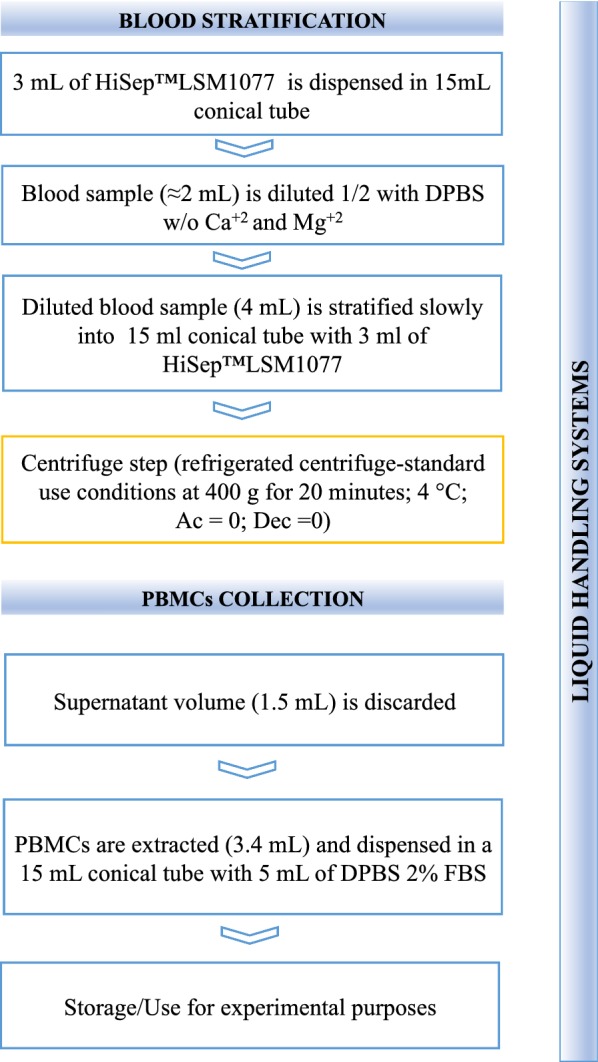
Automatic workflow diagram. Workflow showing the automatic procedures performed by the Freedom EVO 150 for blood stratification and PBMC collection. The blood stratification procedure consists of four necessary steps: (1) density media dispensation; (2) blood sample dilution; (3) diluted blood stratification, and (4) tube recovery for centrifugation (step highlighted in yellow). PBMC collection was performed after centrifugation and consisted of two steps: (1) the removal of 1.5 mL of supernatant and (2) the collection of PBMCs by means of aspirating 3.4 mL of the cellular suspension and subsequent distribution in 15 mL conical tubes containing DPBS + 2% FBS

The script is run following two operating phases described in the workflow diagram presented in Fig. 2 and described as follows:

1. *Blood stratification*: This phase foresees that 3 mL of density gradient medium is distributed at the bottom of 15 mL conical tubes located in the P3 rack (Fig. 1b). The cells residing in the blood pellet of the EDTA tubes (strip rack P1) are gently resuspended in 2 mL of DPBS to obtain a final volume of approximately 4 mL (exploiting two of the four channels on the LiHa arm, 1 mL is added with the 5 mL DiTi and mixed with blood pellet with a 1 mL DiTi for six times, then 1 mL remaining is added again with a 5 mL DiTi and mixed five times with a 1 mL DiTi). This diluted blood solution is then slowly layered onto 3 mL of density gradient media in the P3 rack tubes. At the end of this phase, the tubes are ready for capping and PBMC separation by centrifugation at 400×*g* for 20 min.
2. *PBMC collection*: After centrifugation, the 15 mL conical tubes containing the stratified PBMCs are located in the P3 strip rack. At this moment, 1.5 mL of the supernatant volume is discarded, and a volume of 3.4 mL is collected for distribution and dilution in the corresponding tube containing 5 mL of DPBS positioned in rack P4.

At the end of the second phase, the tubes containing the PBMCs can be used for comparison between manual and automatic procedures and for specific experimental purposes or, alternatively, for storage. Our customized automatic protocol allows processing of 12 samples in one run (45 min automatic procedure/20 centrifugation step).

### Stratification of peripheral blood samples

**Fig. 3:**
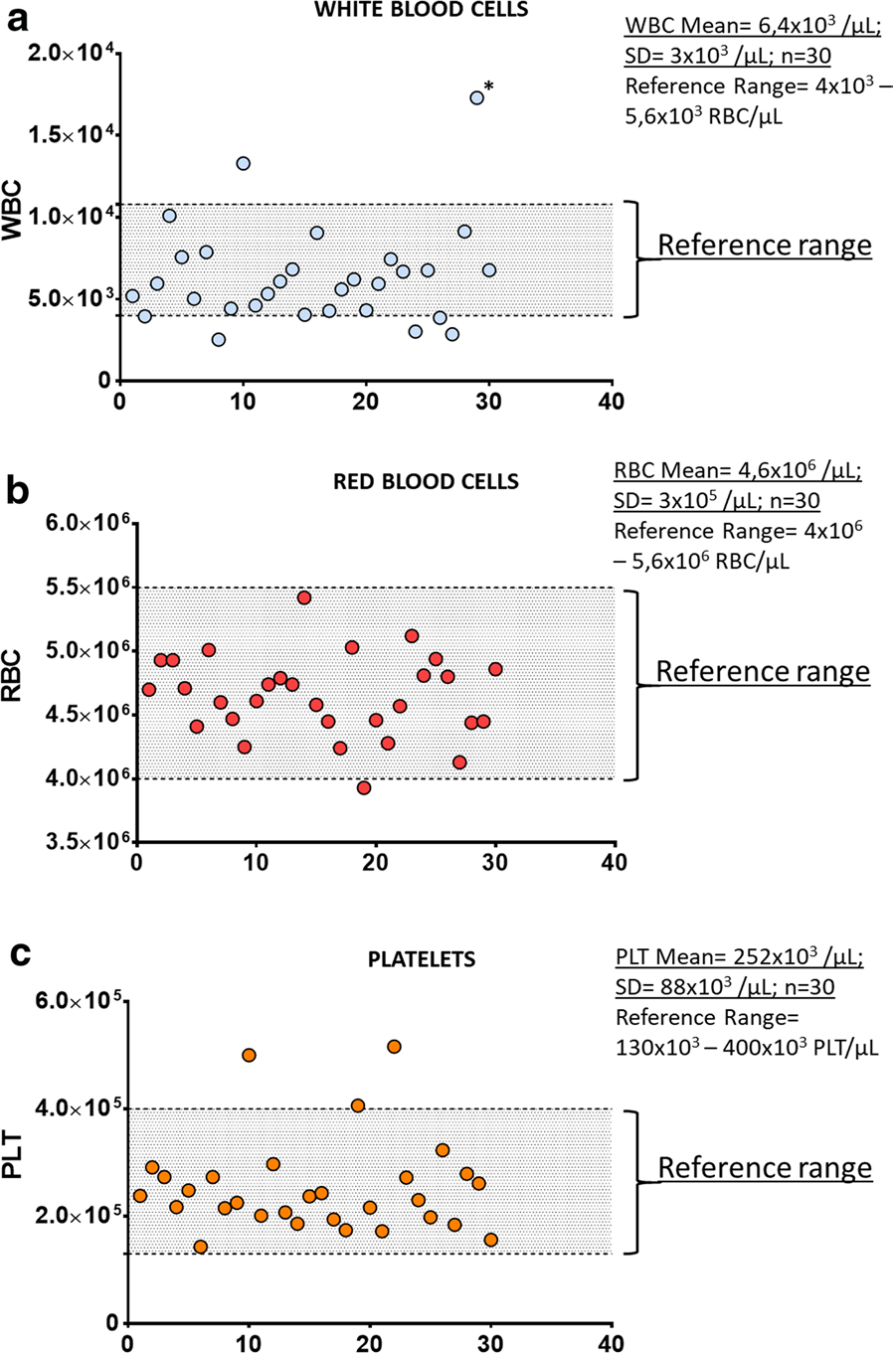
Cellular density of blood cells. The cellular density of white blood cells (WBC), red blood cells (RBC) and platelets (PLT) residing in the peripheral blood of the subjects included in this study are displayed in (**a**–**c)**, respectively. No script errors were highlighted by the workstation during the pipetting of these samples, even in case of a sample with a cellular density of 17 × 10^3^ WBC/µL (marked with *). The normal reference range is highlighted in each graph

The peripheral blood (PB) from 30 consecutive subjects was collected in duplicate in 3 mL EDTA vacutainer test tubes (a total of 60 tubes). A complete blood count was performed before processing each sample. The mean cellular density for white blood cells (WBC), red blood cells (RBC) and platelets (PLT) was of 6.4 × 10^3^/µL (SD = 3 × 10^3^/µL, n = 30), 4.6 × 10^6^/µL (SD = 3 × 10^5^/µL, n = 30) and 252 × 10^3^/µL (SD = 88 × 10^3^/µL, n = 30), respectively. The WBC, RBC and PLT distribution, as well as reference values, are displayed in Fig. 3. During the run of the script, no errors were highlighted, both in the case of stratification and collection of PBMCs. Also, no errors were found when stratification was performed using PB from a patient suffering for an aggressive form of lymphoma where malignant cells were present in the peripheral blood determining a WBC density of 17 × 10^3^ cells/µL. Finally, to better evaluate the functioning of the workstation for stratification of blood samples with high cell density, we processed a peripheral blood sample enriched with HL60 cells to gain a WBC density of about 30 × 10^6^ cells/mL (Additional file 1: Figure S1). The nucleated cellular population, including HL60 cells, was correctly collected, while granulocytes were excluded. These results confirmed the ability of the automatic protocol to process blood samples with a high cellular density as in the case of bone marrow samples.

### Validation of automatic PBMC isolation

**Fig. 4:**
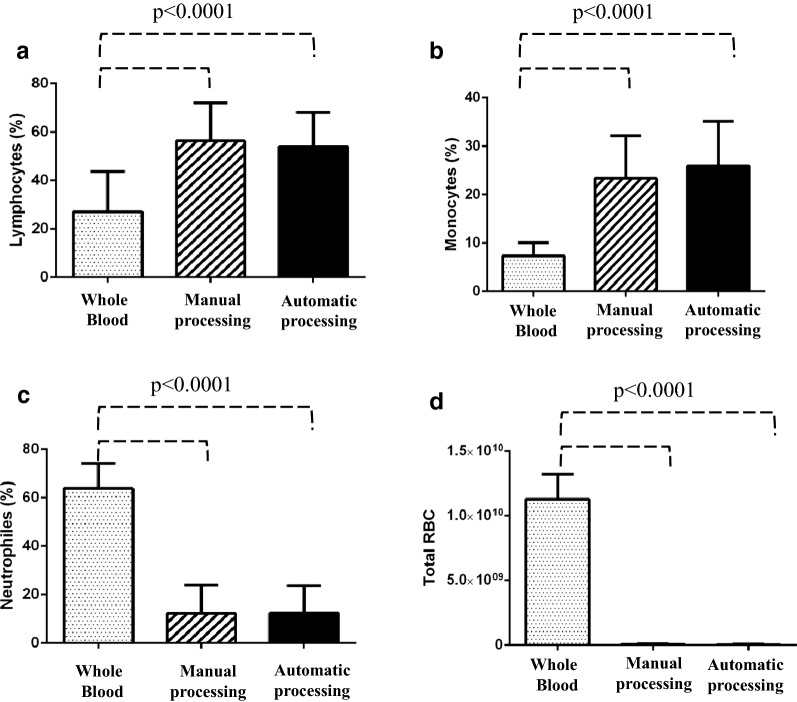
Blood count analysis to compare manual and automatic methods. Bar plot histograms showing the enrichment of PBMCs in terms of the percentage of lymphocytes (**a**) and monocytes (**b**). Additionally, contamination due to the unwanted carryover of neutrophils and red blood cells was significantly reduced (**c**, **d**). The displayed p value was determined using the Mann–Whitney U test with GraphPad Prism software

**Fig. 5:**
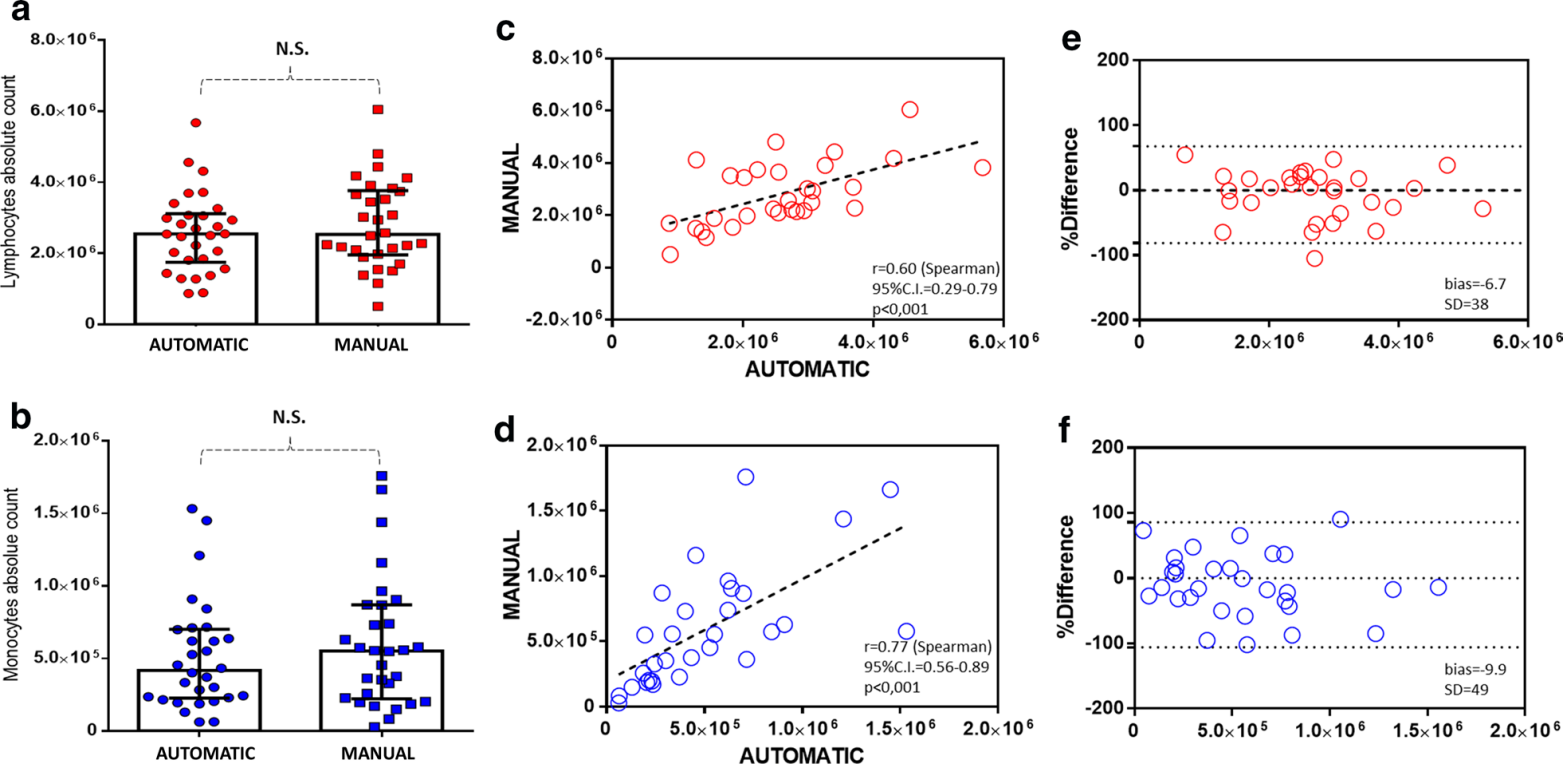
Comparison between manual and automated PBMC isolation procedures. No significant differences (p > 0.05) were detected with regard to the absolute number of recovered lymphocytes and monocytes (**a**, **b**, Mann–Whitney U test). A significant correlation (**c**, **d**, p < 0.001, Spearman test), with bias values of 6.7 and 9.9 (**e**, **f**, Bland–Altman analysis), was recorded for both cellular populations

**Fig. 6:**
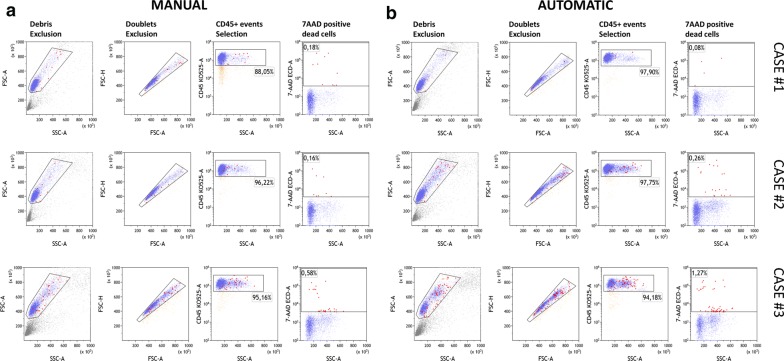
Cell viability determination. Cell viability comparison between manual and automatic PBMC purification. The viability assay using 7AAD (7-ammino-actinomycin D) was performed on three blood samples after manual (**a**) and automatic isolation (**b**). The gating strategy foresees debris and doublets exclusion as describe in FCS-A vs SSC-A and FSC-H vs FSC-A dot-plot, respectively. The cellular population to be tested for 7-AAD staining was selected in terms of CD45 positive events as shown in the CD45-Krome Orange (KO-525) vs SSC-A dot-plot. Dead cells were determined as 7-AAD positive events highlighted in red in the 7-AAD vs SSC-A dot-plot. No significant differences were observed between the two methods (unpaired t-test) as regards the percentage of live cells that was > 98% in all cases

To compare the automatic and manual methods, a complete blood count was performed again after PBMC isolation. In doing so, we were able to evaluate the enrichment for the percentage of lymphocytes and monocytes as well as the carryover due to the unwanted pickup of granulocytes and red cells. As shown in Fig. 4, the blood count analysis showed that both methods were successful (Mann–Whitney test, p < 0.0001) at enriching the percentage of lymphocytes (panel a) and monocytes (panel b) as well as reducing the contamination of neutrophils (panel c) and red blood cells (panel d) in comparison to whole blood. Furthermore, as shown in Fig. 5a, b, no significant differences were detected when comparing the absolute number of total lymphocytes and monocytes isolated using the automatic and manual methods. Additionally, the Spearman correlation test (Fig. 5c, d) and the Bland–Altman plot (Fig. 5e, f) revealed good correlation between the two methods. Indeed, in the case of lymphocytes, the r-value was 0.60 (95% CI 0.29–0.79), and bias was 6.7 (SD = 38), while in the case of monocytes, the r-value was 0.77 (95% CI 0.56–0.89), and bias was − 9.9. To evaluate the cellular viability after PBMC isolation using our automatic method, we included in our study 3 additional consecutive cases. After, PBMC isolation the CD45-Krome Orange (KO)/7-ammino-actinomycin D (7-AAD) stain was performed and samples were analyzed by flow cytometry according to Fig. 6. As expected, no significant differences were found between the manual and automatic protocol. These results showed that the two methods were comparable for the recovery of PBMCs also in terms of viability and prompted us to evaluate their performance in the case of lymphoid subset recovery.

### Preservation of lymphocyte subpopulations by automatic PBMC purification

**Fig. 7:**
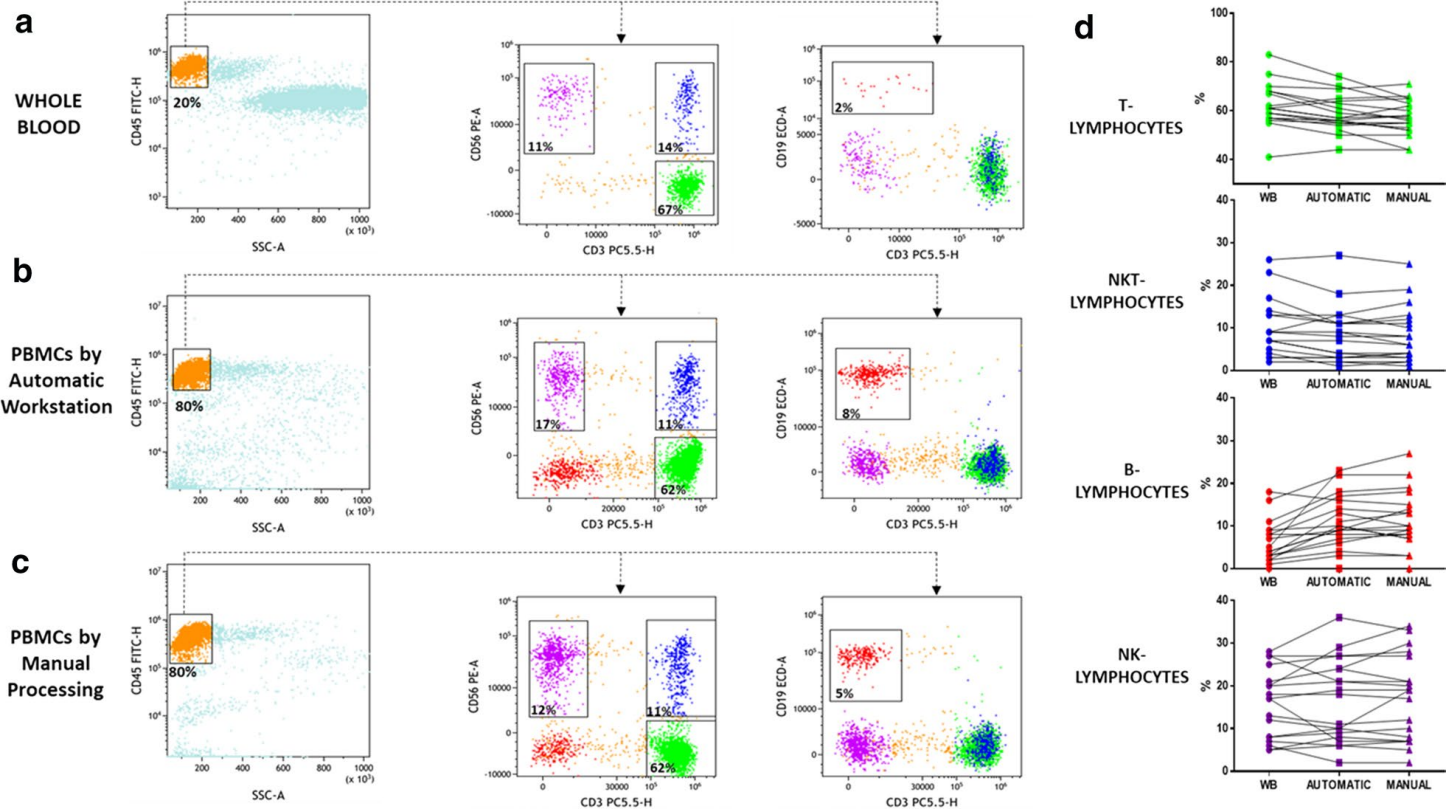
Determination of lymphoid subsets obtained by whole blood, manual and automatic processing. Multicolor flow cytometry analysis shows T, B, NK, and NKT cell percentages in whole blood samples (**a**) and PBMCs derived from the automatic (**b**) and manual (**c**) methods. Left columns show the lymphocyte gating strategy in a CD45 vs SSC dot plot. Middle columns show T, NK and NKT lymphocyte percentages in a CD56 vs CD3 dot plot in terms of CD3^pos^CD56^neg^ (green events), CD3^neg^CD56^pos^ (purple events) and CD3^pos^CD56^pos^ (blue events), respectively. The B lymphocyte percentage was derived as CD19^pos^CD3^neg^ events in the CD19 vs CD3 dot plot (right columns). On the right, the line charts show the before-after graphs to compare the percentage of each lymphocyte subpopulation in whole blood and in PBMCs after automatic and manual separation (**d**)

The preservation of lymphocyte subpopulations is one of the key steps when studying different types of hematological and immunological disorders [15, 16]. To this aim, in a select group of 19 subjects, we evaluated the ability of the automatic stratification method to guarantee correct recovery of the most representative lymphoid subsets. By the use of multicolor flow cytometry analysis, we were able to determine the percentage of T, B, natural killer (NK), and NKT cells in whole blood samples as well as in PBMCs derived either from automatic and manual processing (Fig. 6a–c). Total lymphocytes were selected in a CD45 vs side scatter (SSC) dot plot (Fig. 5a–c), and then T, NK and NKT lymphocyte percentages were determined on a CD56 vs CD3 dot plot (Fig. 6a–c) in terms of CD3^pos^CD56^neg^ (green events), CD3^pos^CD56^pos^ (blue events) and CD3^neg^CD56^pos^ (purple events). The B lymphocyte percentage was derived as CD19^pos^CD3^neg^ events in the CD19 vs CD3 dot plot (Fig. 6a–c). Table 1 shows the percentages of the lymphoid subpopulations detected. Interestingly, compared with whole blood, we found that the percentages of lymphoid subsets were similar except in the case of B cells, which were present at a higher percentage after density gradient stratification (Fig. 6d). Furthermore, to compare the automated and manual sample processing methods, we performed Bland–Altman and Spearman correlation tests, as displayed in Fig. 7a–d. As expected, for T, B, NKT and NK lymphocytes, a significant (p < 0.0001) correlation was found, with r (Spearman) values of 0.90 (95% CI 0.74–0.96), 0.87 (95% CI 0.68–0.95), 0.97 (95% CI 0.93–0.99), and 0.96 (95% CI 0.89–0.98), respectively; Bland–Altman analysis revealed bias values of 2.85 (SD = 6.4),− 0.67 (SD = 22.3), − 2.74 (SD = 18.6), and 1.1 (SD = 28.3), respectively.

**Table 1:** Lymphoid subpopulations detected in whole blood, and PBMCs from automatic and manual processing

Sample ID	Sample processing	T-cells (CD3+/CD56−) (%)	B-cells (CD19+/CD3−) (%)	NK-cells (CD3−/CD56+) (%)	NKT (CD3+/CD56+) (%)
1	Wb	57	3	27	9
	Automatic	56	6	27	8
	Manual	52	9	27	8
2	Wb	82	8	5	2
	Tecan	76	14	7	2
	Manual	77	13	7	2
3	Wb	80	4	5	9
	Automatic	69	9	8	13
	Manual	71	10	7	10
4	Wb	61	2	20	7
	Automatic	57	4	29	4
	Manual	58	3	34	3
5	Wb	63	8	15	5
	Automatic	52	11	24	9
	Manual	44	13	30	6
6	Wb	62	9	18	3
	Automatic	65	10	18	3
	Manual	66	7	17	3
7	Wb	41	18	21	17
	Automatic	44	16	24	11
	Manual	44	15	21	13
8	Wb	68	11	7	13
	Automatic	63	18	6	11
	Manual	60	19	7	12
9	Wb	55	8	25	7
	Tecan	50	8	27	7
	Manual	50	8	28	6
10	Wb	83	5	6	4
	Automatic	74	23	1	1
	Manual	65	27	2	2
11	Wb	59	16	17	5
	Automatic	55	22	19	3
	Manual	55	22	19	4
12	Wb	57	0	28	2
	Automatic	54	0	36	2
	Manual	55	0	33	1
13	Wb	56	9	5	23
	Automatic	55	17	6	18
	Manual	53	18	5	19
14	Wb	68	2	12	14
	Automatic	61	10	10	11
	Manual	56	7	19	11
15	Wb	61	1	8	26
	Automatic	57	3	10	27
	Manual	62	3	8	25
16	Wb	75	4	8	9
	Automatic	70	9	9	9
	Manual	63	14	10	8
17	Wb	59	7	20	7
	Automatic	56	9	21	4
	Manual	58	9	20	4
18	Wb	61	3	27	3
	Automatic	64	7	21	3
	Manual	65	8	21	4
19	Wb	67	3	13	13
	Automatic	59	13	11	13
	Manual	57	10	12	16

## Discussion

The isolation of mononuclear cells from peripheral blood is a protocol routinely used in biobanking [17]. Notably, PBMCs include a heterogeneous population of cells that are mainly involved in the body’s immune defense, as in the case of B, T, NK and NKT lymphocytes, monocytes and dendritic cells [18]. The study of these immune cell subsets can be of interest in different fields of medical research. For example, T and B cells have been shown to be activated during neuroinflammatory autoimmune diseases such as Parkinson’s disease [19] and multiple sclerosis [20]; circulating tumor cells have been detected in cryopreserved PBMCs from patients affected with sarcoma [21] and are intensively studied in breast, lung, prostate and colorectal cancers [22]. Despite the broad field of applications, the isolation of PBMCs from peripheral blood is a laboratory procedure still affected by high operator-dependent variability. The dilution, stratification, and collection of mononuclear cells from the density gradient medium are some of the critical steps that affect standardization. Over time, different attempts have been made to standardize the PBMC isolation procedure; however, few reports were able to present the automatization of the entire procedure. In 2012, Mathay et al. [23] developed an automated process with the Tecan pipetting robot Freedom EVO 200 for extraction of the buffy coat (BC) from three subjects. The comparison with the automated method was performed by means of a complete blood count and DNA extraction. The authors combined BC extraction with upstream automated plasma aliquoting and downstream DNA extraction from BC in the same run. Regardless of the low number of subjects (n = 3) examined in their work, they were able, for the first time, to optimize the manual procedure for BC extraction on the Tecan system. In 2015, Hamot et al. [24] improved the workflow using the Tecan system again for the automated isolation of PBMCs from whole blood collected in BC CPT vacutainer tubes (citrate anticoagulant). The authors validated the automated procedure in terms of cell yield, viability, recovery, white blood cell subpopulation distribution, gene expression, and lymphoblastoid cell line transformation. Interestingly, they compared the performance of CPT tubes in isolating PBMCs with respect to the manual procedure performed using Ficoll^®^ and Leucosep^®^.

In the present paper, in contrast to Hamot et al. [24], we developed an automated script for the isolation of PBMCs from whole blood collected in K3 EDTA vacutainer tubes using density gradient media (HiSep). In comparison to the work of Hamot et al., we did not use CPT tubes for the setup of the automated procedure to make the entire procedure cost-saving and feasible in all laboratories equipped with a robotized workstation similar to the Tecan Freedom EVO 150. The automatic procedure was validated by comparison to the manual procedure. Under our experimental conditions, we demonstrated that automation guarantees the same yields as the manual procedure in terms of the number of recovered lymphocytes and monocytes and the reduced contamination of neutrophils and red blood cells.

Furthermore, no significant differences were found between the two methods in terms of the percentages of lymphocyte subpopulations. The automatic separation of PBMCs also guarantees no operator-related variability, the identification and tracking of the sample in all protocol phases, and the recording of all instrument-specific parameters during isolation. Additionally, we ascertained that automatic processing guarantees sterile biological samples. In fact, after the automated procedure, isolated PBMCs were grown in complete medium for up to 10 days, and no contamination was observed (data not shown). Finally, it is important to consider that using the automatic workflow, it is possible to develop additional upstream or downstream procedures, such as the generation of plasma aliquots and the isolation of nucleic acids, respectively. The generation and optimization of automatic protocols using robotized liquid handling systems will be useful for ameliorating the standardization of biobanking procedures [25].

In our study, we presented an automatic process related to the isolation of PBMCs from peripheral blood in the workflow of our biobank. It has considerable advantages as mentioned above; however, technical and practical limitations are present. The first aspect concerns the lack of a blood level detection unit. Our automatic protocol was developed on the basis of spatial coordinates x, y, and z, in which the use of fixed volumes determine the specific spatial coordinate of the phase in which the PBMCs are present. For the right set-up of the x, y and z coordinates we were assisted by the manufacturer technical support both on-site (for the instrument pipeline set-up) and online (for the script development). During the blood stratification process, it was important to generate a homogeneous cellular suspension to be gently stratified over the density gradient media. This difficult problem was resolved by programming the instrument for doing the following operations: (1) add 1 mL of DPBS; (2) mix up and down for five times; (3) add a second 1 mL volume of DPBS; (4) mix up and down for five times; (5) immediately distribute over density gradient media. Also, during blood stratification, the flow rate was imposed to be as slow as possible (20 µL/s–1 mL Tip) for avoiding mixing of the diluted blood with the density media, especially during the first steps of stratification. Importantly, additional instrument components (still not available in our case) for script optimization could be useful. For example, the installation of a laser-based inspection unit for a more accurate determination of the x, y and z coordinates could be helpful, especially for the blood stratification as well as for identification and picking-up of the PBMC layer after centrifugation. The setting-up of this technical procedure required about eight months of internal test and close collaboration with the specialized instrumentation technician provided by the manufacturer. Subsequently, a two-week training period was necessary for all biobank operators to learn practical information (relating to the workstation such as replacing the tip slots, reagents, correct placement of blood samples in the loading position) and information relating to the machine management software. Currently, all the operators of our biobank are able to manage the instrument easily. Another critical aspect concerns the time needed for processing the blood samples; currently, our automatic script takes 45 min to process (from dilution to stratification) 12 blood samples.

Finally, although automation represents an approach used by many laboratories around the world, the costs related to instruments and consumables remain inaccessible to infrastructures with limited funds, especially in developing countries.

## Conclusions

Our manuscript demonstrates that the automated isolation of PBMCs is feasible with an automated system and comparable to traditional manual procedures; we believe that automation is becoming essential in the biomedical and diagnostics research field because it will allow biobank networks to standardize biological sample processing, allowing a fair comparison of biological data analyses downstream. In this way, it will be possible to reach the overall goal of an international research framework that aims to facilitate access to human biological materials. The life cycle stages of biosamples [26] (intended as the collection, accession, acquisition, identification, preservation, long-term storage, quality control (QC), transport, and disposal of biomaterials) are a major source of heterogeneity, and process automation could represent a potential means of addressing these obstacles.

## Methods

### Study population

We selected a group of 33 adult subjects of both sex, 13 females and 20 males, with an average age of 62.6 (range, years 33–87), enrolled at the SDN biobank in Naples (protocol number: 7 approved on 22/07/2015 by the ethical committee of IRCCS Pascale, Naples). All patients signed informed consent for biobanking their samples. All laboratory equipment for analyses was calibrated, and the traceability of biobanking workflow was guaranteed as recommended by the ISO 9001 document.

### Samples processing

**Fig. 8:**
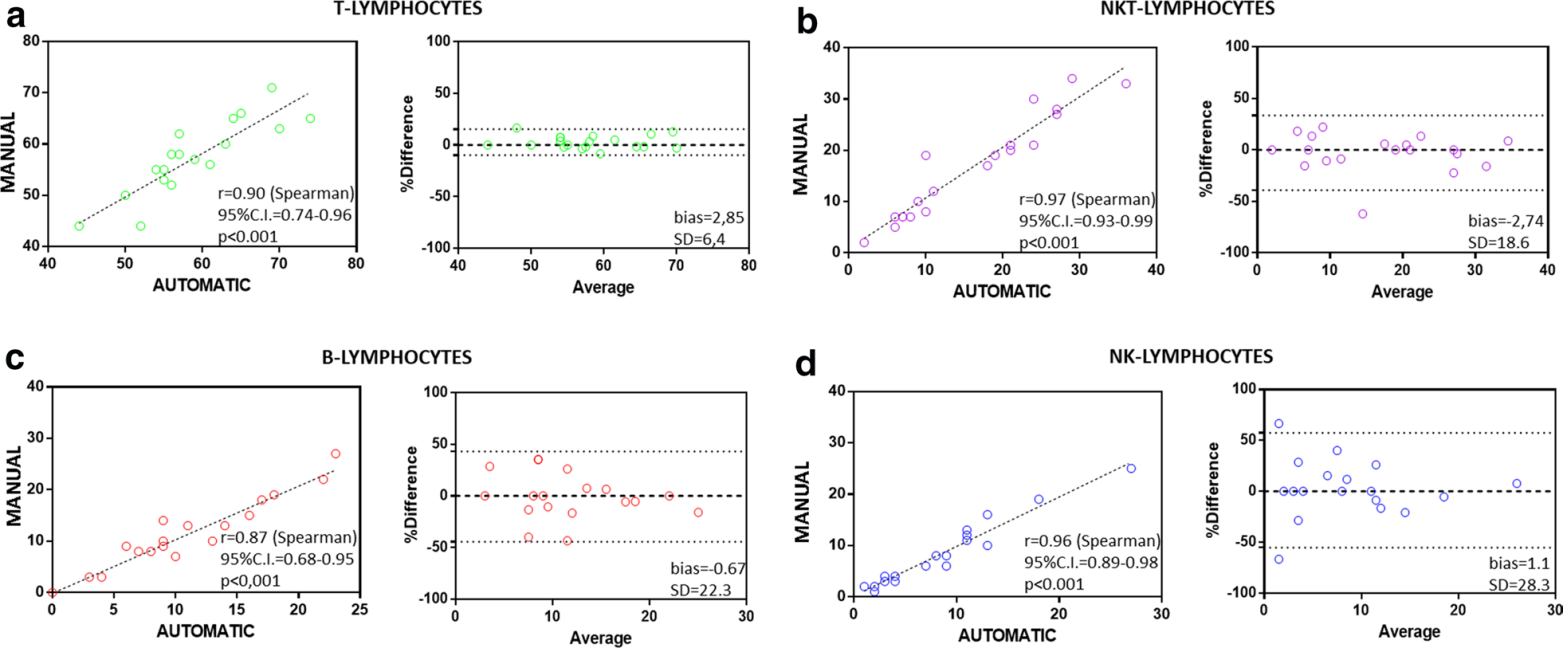
Bland–Altman and Spearman correlation tests of lymphoid subsets. Bland–Altman and Spearman correlation tests are displayed (**a**–**d**). T, B, NKT and NK-lymphocyte subsets were recovered, with a significant (p < 0.0001) correlation between the automatic and manual methods according to the r (Spearman) values of 0.90 (95% CI 0.74–0.96), 0.87 (95% CI 0.68–0.95), 0.97 (95% CI 0.93–0.99), and 0.96 (95% CI 0.89–0.98), respectively. Bland–Altman analysis revealed bias values of 2.85 (SD = 6.4), − 0.67 (SD = 22.3), − 2.74 (SD = 18.6), and 1.1 (SD = 28.3), respectively

All samples were collected in 3 mL K3-EDTA vacutainer tubes (Becton–Dickinson, Ref. 368860), mixed immediately after the collection by inverting 10 times, and shipped to the lab in less than 30 min. Complete blood count was performed using the ADVIA 120 Hematology System (Siemens Healthcare); lymphocytes subpopulation analysis was performed using the CytoFLEX (Beckman Coulter) flow cytometer after staining with CD45FITC-CD56PE-CD19ECD-CD3PC5 antibody mix (Beckman Coulter #6607073). For each patient enrolled, human whole blood (500 µl) and PBMCs isolated with manual and automatic procedures were studied. All the operators in the study were well trained according to a Standard Operating protocol and randomly selected for sample processing. The cellular viability was determined by means of staining PBMC with 7-AAD (Beckman Coulter, #IM3422) and CD45-KO (Beckman-Coulter, #B36294). Gating strategy is described in Fig. 8, debris was excluded according to the FSC-A vs SSC-A analysis. Then, single cells were selected according to FSC-Height vs FSC-Area dot-plot, and CD45 positive cells were identified on the CD45 vs SSC-A dot-pot. Dead cells were determined as the percentage of 7-AAD positive events according to the 7AAD vs SSC dot plot.

### HL60 enriched blood sample

To simulate a blood sample with high cell density, such as bone marrow samples and/or blood samples obtained by patients with proliferative hematological diseases, we spiked 120 × 10^6^ HL60 cells in 4 mL blood sample. This blood sample was characterized using the CytoFLEX (Beckman Coulter) flow cytometer before and after automatic isolation using CD45KO (Beckman Coulter #B36294) and CD33PE (Beckman Coulter #A07775) antibodies. HL60 cells were grown in RPMI complete medium supplemented with 10% of fetal bovine serum (FBS) and 1% Glutamax (Thermo Fisher Scientific, GIBCO, #35050) at 37 °C and 5% CO_2_. HL60 cell line was provided by SDN Biobank and validated at DSMZ according to STR profile.

### Manual PBMCs isolation

Centrifuged and plasma separated whole blood EDTA was used for the purification of PBMCs. The manual protocol for the density gradient separation of PBMCs began with the dispensation of 3 mL of HiSep™ LSM1077 (HiMedia Laboratories, Mumbai, India) in a conical sterile centrifuge tube. Then, peripheral blood was diluted 1/2 in Dulbecco’s phosphate-buffered saline (DPBS) solution w/o Ca^2+^ and Mg^2+^ (Sigma-Aldrich, MO, USA), stratified onto 3 mL of density gradient media and centrifuged at 400×*g* for 20 min and 4 °C. After centrifugation and using a sterile pipette, the PBMC layer was transferred to a new 15 mL centrifuge tube and diluted in DPBS supplemented with 2% fetal bovine serum (FBS). Two wash steps were performed before starting the controlled − 1 °C/min freeze storage procedure or using the PBMCs for experimental procedures.

### Consumables for automatic PBMC isolation

In detail, the racks and consumables used for the Tecan Freedom EVO 150 work platform are as follows: (i) rack strip, 16 positions, tube 13 mm, #30019986; (ii) carrier Falcon tube, 15 mL, 12 positions, #30012505; (iii) carrier Falcon tube, 50 mL, tube 8 positions, #30012708; (iv) DITI LIHA, 1000 μL, #30000631; (v) DITI LIHA, 5000 μL, #30059898, provided by Tecan Italia; (vi) gradient density (HiSep™ LSM1077 sterile-filtered, #10771, HiMedia Laboratories, Mumbai, India); (vii) DPBS, #14190250, Gibco; and (viii) FBS, Gibco, #10270106.

### Statistical analysis

The statistical analysis performed for comparing the two methodologies is described in the results section. Differences between groups were considered to be significant at a p value of < 0.05. Statistical analyses were performed with GraphPad Prism 6.0 (GraphPad Software, Inc., San Diego, CA).

## Supplementary information

**Additional file 1: Figure S1** Purification of mononuclear cells from a peripheral blood sample enriched with HL60 cells to gain a WBC cell density of 30 × 106 cells/mL. HL60 (blue), mononuclear cells (red) and granulocytes (green)were detectable according to the FSC-A vs SSC-A dot-plot. Granulocytes’ population is lost after PBMC automatic isolation, while the percentage of HL60 and mononuclear cells were enriched. CD45 vs CD33 dot-plot is shown to better identify the HL60 cells that derive from CD33 positive human acute myeloid leukemia (M2).

## Data Availability

The datasets generated and/or analyzed during the current study are available at SDN Biobank. The datasets used and analyzed during the current study are available from the corresponding author (Biobank Curator, Dr. Peppino Mirabelli, PhD; mail: pmirabelli@sdn-napoli.it) on reasonable request.

## Abbreviations

BBMRI-ERIC: BioMolecular Resources Research Infrastructure—European Research Infrastructure Consortium
DPBS: Dulbecco’s phosphate-buffered saline
EDTA: ethylenediaminetetraacetic acid
FBS: fetal bovine serum
NK: natural killer
PBMCs: peripheral blood mononuclear cells
PLT: platelets
RBC: red blood cells
SOPs: standard operating procedures
WBC: white blood cells
7AAD: 7-ammino-actinomycin D
