# Supplementary material for: Purification of viable peripheral blood mononuclear cells for biobanking using a robotized liquid handling workstation
Source: J Transl Med. 2019 Nov 12;17:371. doi: 10.1186/s12967-019-2125-7 (PMC6852781; doi:10.1186/s12967-019-2125-7)
Supplement: Supplementary file 1 — Additional file 1: Figure S1 Purification of mononuclear cells from a peripheral blood sample enriched with HL60 cells to gain a WBC cell density of 30 × 106 cells/mL. HL60 (blue), mononuclear cells (red) and granulocytes (green)were detectable according to the FSC-A vs SSC-A dot-plot. Granulocytes’ population is lost after PBMC automatic isolation, while the percentage of HL60 and mononuclear cells were enriched. CD45 vs CD33 dot-plot is shown to better identify the HL60 cells that derive from CD33 positive human acute myeloid leukemia (M2). [file 12967_2019_2125_MOESM1_ESM.pdf]

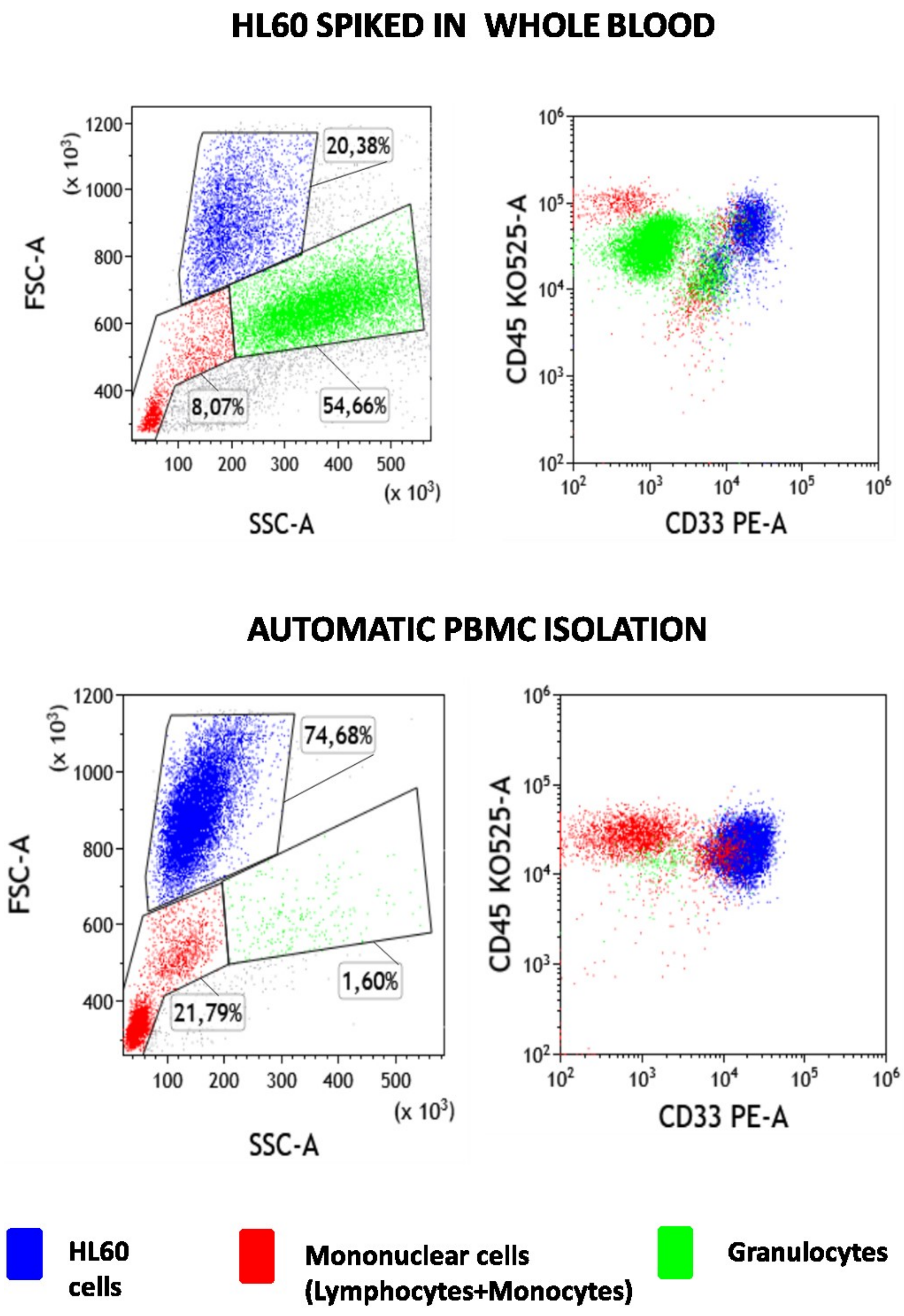

Additional file 1: Figure S1. Purification of mononuclear cells from a peripheral blood sample enriched with HL60 cells to gain a WBC cell density of $30x10^6$ cells/mL. HL60 (blue), Mononuclear cells (red) and Granulocytes (green)were detectable according to the FSC-A vs SSC-A dot-plot. Granulocytes' population is lost after PBMC automatic isolation, while the percentage of HL60 and mononuclear cells were enriched. CD45 vs CD33 dot-plot is shown to better identify the HL60cells that derive from CD33 positive human acute myeloid leukemia (M2).
